# Supplementary material for: Cardiovascular Disease Burden Attributable to High Sodium Intake in China: A Longitudinal Study from 1990 to 2019
Source: Nutrients. 2024 Apr 26;16(9):1307. doi: 10.3390/nu16091307 (PMC11085757; doi:10.3390/nu16091307)
Supplement: Supplementary file 1 [file nutrients-16-01307-s001.zip › Supplementary Table S1.pdf]

Supplementary Table S1 Codes in (ICD-10) for major CVD subcategories.

1	cardiomyopathy and myocarditis (B33.2, I40-I41.9, I42.1-I42.8, I43-I43.9, I51.4);
2	non-rheumatic valvular heart disease (I34-I37.9);
3	atrial fibrillation and flutter (I48-I48.9);
4	endocarditis (I33-I33.9, I38-I39.9);
5	stroke (G45-G46.8, I60-I62.9, I63-I63.9, I65-I66.9, I67.2-I67.3, I67.5-I67.6, I67.0-I67.1, I68.1-I68.2, I69.0-I69.2, I69.3);
6	rheumatic heart disease (I01-I01.9, I02.0, I05-I09.9);
7	hypertensive heart disease (I11-I11.9);
8	ischemic heart disease (I20-I25.9);
9	aortic aneurysm (I71-I71.9);
10	peripheral vascular disease (I70.2-I70.8, I73-I73.9);
11	other cardiovascular and circulatory diseases (the rest of the CVD codes).
