# Supplementary material for: Cardiovascular Disease Burden Attributable to High Sodium Intake in China: A Longitudinal Study from 1990 to 2019
Source: Nutrients. 2024 Apr 26;16(9):1307. doi: 10.3390/nu16091307 (PMC11085757; doi:10.3390/nu16091307)
Supplement: Supplementary file 1 [file nutrients-16-01307-s001.zip › Supplementary Table S2.pdf]

Supplementary Table S2. Deaths and ASMR of cardiovascular disease attributable-high sodium intake in 1990 and 2019 and the temporal trends from 1990-2019.

Characteristics	1990		2019			1990-2019
	Deaths cases, No. (95% UI)	ASMR per 100,000 No. (95% UI)	Deaths cases, No. (95% UI)	ASMR per 100,000 No. (95% UI)	PAFs % (95% UI)	EAPC (%) in ASMR No. (95% CI)
**Region**						
Anhui	28300.82 (14713.35 to 44312.37)	81.82 (38.37 to 134.66)	42365.07 (16674.31 to 75509.82)	49.64 (18.75 to 89.83)	0.18 (0.07 to 0.31)	-1.48 (-1.63 to -1.33)
Beijing	7488.02 (3862.88 to 11437.14)	90.30 (43.45 to 144.21)	8999.36 (3470.48 to 16079.41)	28.94 (10.47 to 52.37)	0.16 (0.06 to 0.29)	-4.47 (-4.78 to -4.17)
Chongqing	6186.35 (2473.33 to 10825.82)	57.01 (19.68 to 105.62)	15689.27 (5177.91 to 30054.07)	38.24 (12.06 to 74.8)	0.15 (0.05 to 0.27)	-1.00 (-1.17 to -0.83)
Fujian	10179.05 (4474.88 to 17345.06)	57.35 (22.84 to 102.39)	12682.98 (4233.43 to 23787.90)	27.77 (8.67 to 53.34)	0.14 (0.05 to 0.27)	-2.50 (-2.7 to -2.29)
Gansu	3482.44 (580.02 to 7834.64)	27.86 (3.75 to 67.23)	9058.00 (1805.78 to 19719.91)	28.35 (4.99 to 66.32)	0.09 (0.02 to 0.19)	0.56 (0.37 to 0.75)
Guangdong	19397.98 (6396.47 to 36491.31)	47.25 (13.88 to 94.46)	26757.60 (7666.72 to 53343.85)	21.74 (5.62 to 45.16)	0.11 (0.03 to 0.23)	-1.77 (-2.05 to -1.49)
Guangxi	12028.01 (3673.07 to 22895.45)	44.93 (12.21 to 90.07)	18553.34 (4012.57 to 39340.86)	31.56 (6.42 to 68.75)	0.10 (0.02 to 0.22)	-1.77 (-2.05 to -1.49)
Guizhou	14604.43 (6794.08 to 24247.93)	75.21 (31.95 to 129.38)	20888.09 (7684.4 to 38367.14)	49.77 (16.99 to 94.15)	0.16 (0.06 to 0.28)	-1.23 (-1.34 to -1.12)
Hainan	1775.32 (530.04 to 3489.34)	43.42 (11.63 to 88.71)	2918.63 (687.46 to 6069.51)	28.24 (5.94 to 61.34)	0.10 (0.02 to 0.21)	-1.30 (-1.54 to -1.07)
Hebei	38646.85 (17548.55 to 63192.08)	93.83 (38.79 to 158.93)	67766.87 (27985.58 to 115833.93)	70.79 (26.25 to 128.23)	0.18 (0.07 to 0.3)	-0.87 (-1.04 to -0.70)
Heilongjiang	17793.13 (7599.22 to 29396.77)	95.22 (34.91 to 167.84)	28523.97 (9065.77 to 54018.82)	50.31 (13.86 to 103.86)	0.12 (0.03 to 0.24)	-1.98 (-2.21 to -1.74)

Henan	41009.79 (18848.89 to 66550.96)	73.07 (30.18 to 124.8)	65058.13 (25447.86 to 115571.80)	55.23 (20.22 to 101.41)	0.16 (0.06 to 0.29)	-0.60 (-0.72 to -0.48)
Hong Kong	1654.80 (696.50 to 2806.60)	31.67 (12.16 to 55.75)	2150.85 (660.92 to 4439.26)	13.90 (4.64 to 27.92)	0.15 (0.05 to 0.27)	-2.83 (-3.12 to -2.53)
Hubei	30182.37 (13854.17 to 50146.24)	87.37 (35.47 to 152.3)	32773.79 (10915.76 to 60719.68)	41.11 (12.28 to 79.43)	0.14 (0.04 to 0.27)	-2.33 (-2.56 to -2.11)
Hunan	25800.85 (10130.72 to 46295.77)	64.47 (22.52 to 122.3)	44372.51 (15393.87 to 82953.04)	48.11 (15.35 to 93.57)	0.15 (0.05 to 0.28)	-0.68 (-0.80 to -0.56)
Inner Mongolia	11508.91 (5534.85 to 18402.90)	100.51 (43.61 to 167.96)	21194.01 (9584.76 to 35084.53)	66.35 (26.43 to 116.65)	0.18 (0.08 to 0.31)	-1.22 (-1.39 to -1.05)
Jiangsu	24574.19 (10732.36 to 40684.44)	50.46 (20.21 to 87.43)	33128.31 (11795.37 to 61699.68)	25.50 (8.48 to 48.85)	0.15 (0.05 to 0.27)	-2.42 (-3.21 to -1.63)
Jiangxi	21327.18 (11264.71 to 32675.02)	96.36(46.49 to 154.65)	24731.19 (10492.98 to 42585.82)	47.34 (17.99 to 85.09)	0.18 (0.07 to 0.31)	-2.42 (-3.21 to -1.63)
Jilin	19922.32 (10410.24 to 30111.75)	139.35(65.41 to 219.91)	22891.75 (8730.61 to 40199.96)	57.18 (19.34 to 104.61)	0.15 (0.05 to 0.28)	-3.23 (-3.52 to -2.94)
Liaoning	17148.34 (6038.51 to 30681.95)	65.85 (20.13 to 124.13)	32866.13 (10563.84 to 61932.40)	44.78 (13.11 to 88.38)	0.13 (0.04 to 0.25)	-1.05 (-1.25 to -0.86)
Macao	107.38 (44.53 to 186.84)	40.42 (16.3 to 70.67)	158.76 (56.00 to 290.29)	16.90 (5.64 to 31.78)	0.16 (0.06 to 0.29)	-3.07 (-3.22 to -2.92)
Ningxia	1039.23 (302.09 to 2053.28)	48.40 (11.58 to 101.92)	2681.04 (813.50 to 5203.30)	39.82 (9.90 to 82.16)	0.11 (0.03 to 0.23)	-0.32 (-0.47 to -0.18)
Qinghai	1770.85 (923.08 to 2764.53)	85.03 (38.57 to 141.91)	4081.31 (1935.00 to 6686.12)	71.14 (30.04 to 124.28)	0.18 (0.08 to 0.31)	-0.28 (-0.50 to -0.05)
Shaanxi	17131.53 (7980.26 to 27969.13)	85.14 (36.69 to 144.93)	30081.75 (12621.71 to 51956.40)	61.59 (23.25 to 111.58)	0.17 (0.07 to 0.30)	-0.76 (-0.93 to -0.59)
Shandong	41378.98 (19401.39 to 68828.63)	70.99 (30.02 to 124.08)	65741.60 (25815.99 to 116443.20)	45.88 (16.51 to 84.24)	0.16 (0.06 to 0.29)	-1.32 (-1.42 to -1.22)
Shanghai	5838.51 (2415.15 to 9885.65)	46.48 (17.62 to 82.23)	6994.25 (2249.26 to 13347.51)	16.91 (5.31 to 33.04)	0.13 (0.04 to 0.24)	-3.85 (-4.19 to -3.50)
Shanxi	10806.46 (3230.82 to 20782.59)	58.18 (14.97 to 118.87)	21076.38 (7154.15 to 39994.57)	46.91 (14.05 to 92.7)	0.13 (0.04 to 0.25)	-0.62 (-0.75 to -0.49)

Sichuan	37507.45 (15097.31 to 66574.98)	52.36 (18.57 to 97.78)	47068.70 (16000.22 to 88296.43)	38.02 (11.97 to 73.70)	0.14 (0.05 to 0.27)	-0.61 (-0.89 to -0.33)
Tianjin	3834.44 (1461.56 to 6714.63)	57.76 (19.23 to 108.08)	6375.62 (1827.69 to 12531.96)	31.71 (8.27 to 65.21)	0.12 (0.03 to 0.23)	-1.81 (-2.18 to -1.43)
Tibet	2134.64 (1084.30 to 3392.78)	159.09(73.14 to 265.75)	2579.39 (1312.44 to 4030.69)	105.86 (48.01 to 174.27)	0.23 (0.11 to 0.38)	-1.55 (-1.66 to -1.44)
Xinjiang	6307.47 (2593.9 to 10933.50)	80.57 (28.15 to 151.48)	18811.71 (8988.6 to 30966.61)	82.32 (35.03 to 142.37)	0.20 (0.08 to 0.33)	0.38 (0.26 to 0.50)
Yunnan	12453.30 (4872.16 to 21773.45)	54.27 (19.05 to 99.89)	24980.07 (9513.89 to 43680.73)	47.63 (17.00 to 87.60)	0.15 (0.05 to 0.27)	-0.15 (-0.38 to 0.08)
Zhejiang	19025.02 (10072.54 to 29981.56)	62.32 (31.1 to 102.02)	24587.34 (10418.25 to 41109.62)	29.81 (12.10 to 50.79)	0.20 (0.08 to 0.33)	-2.51 (-2.67 to -2.35)

ASMR=age-standardized mortality rate; PAF=population attributable fraction; EAPC=estimated annual percentage change.
