# Supplementary material for: Cardiovascular Disease Burden Attributable to High Sodium Intake in China: A Longitudinal Study from 1990 to 2019
Source: Nutrients. 2024 Apr 26;16(9):1307. doi: 10.3390/nu16091307 (PMC11085757; doi:10.3390/nu16091307)
Supplement: Supplementary file 1 [file nutrients-16-01307-s001.zip › Supplementary Table S3.pdf]

Supplementary Table S3. DALYs and ASDR of cardiovascular disease attributable to high sodium intake in 1990 and 2019 and the temporal trends from 1990 to 2019.

Characteristics	1990		2019			1990-2019
	DALYs, No. (95% UI)	ASDR per 100,000 No. (95% UI)	DALYs, No. (95% UI)	ASDR per 100,000 No. (95% UI)	PAFs % (95% UI)	EAPC (%) in ASDR No. (95%CI)
**Region**						
Anhui	784687.49 (439424.66 to 1194778.43)	1877.69 (1003.27 to 2949.34)	965362.92 (452977.83 to 1583057.23)	1050.93 (476.39 to 1750.82)	0.22 (0.11 to 0.35)	-1.84 (-1.99 to -1.69)
Beijing	196837.90 (110004.61 to 293424.22)	2020.96 (1078.80 to 3068.81)	218552.07 (98356.13 to 365147.55)	644.29 (280.95 to 1085.94)	0.20 (0.09 to 0.33)	-4.51 (-4.81 to -4.21)
Chongqing	175320.69 (78385.51 to 292366.38)	1375.81 (578.54 to 2399.12)	352932.95 (138365.73 to 625756.99)	829.16 (328.38 to 1463.14)	0.19 (0.08 to 0.31)	-1.45 (-1.59 to -1.32)
Fujian	274400.79 (131768.17 to 446562.40)	1326.42 (612.88 to 2226.28)	295855.24 (123756.42 to 509849.90)	580.37 (230.91 to 1023.14)	0.18 (0.08 to 0.31)	-2.96 (-3.16 to -2.76)
Gansu	106165.36 (20870.77 to 227064.97)	693.91 (118.38 to 1554.89)	233550.70 (55571.71 to 472678.18)	654.57 (153.17 to 1364.99)	0.12 (0.03 to 0.23)	0.18 (0.02 to 0.34)
Guangdong	509653.86 (193769.60 to 908953.71)	1082.12 (387.38 to 1969.90)	686032.66 (254468.70 to 1239650.26)	502.53 (174.55 to 928.91)	0.15 (0.05 to 0.27)	-1.89 (-2.14 to -1.63)
Guangxi	320525.41 (113166.21 to 572199.21)	1064.33 (357.80 to 1965.34)	439015.16 (111311.49 to 885682.85)	701.96 (173.87 to 1426.64)	0.13 (0.03 to 0.25)	-1.89 (-2.14 to -1.63)

Guizhou	408514.43 (204242.30 to 652375.73)	1843.36 (894.08 to 3011.94)	516714.27 (224459.74 to 892181.67)	1143.78 (486.75 to 2002.76)	0.20 (0.09 to 0.32)	-1.52 (-1.59 to -1.45)
Hainan	47388.13 (16561.26 to 88541.34)	1018.95 (333.74 to 1948.12)	73470.42 (21611.4 to 142275.22)	638.97 (176.18 to 1271.88)	0.13 (0.04 to 0.25)	-1.48 (-1.67 to -1.29)
Hebei	999755.77 (510453.21 to 1552767.06)	2109.25 (1025.67 to 3375.17)	1713053.26 (815310.07 to 2730597.14)	1609.99 (721.61 to 2644.41)	0.22 (0.10 to 0.35)	-0.92 (-1.06 to -0.79)
Heilongjiang	534545.75 (249088.11 to 861411.95)	2307.04 (991.13 to 3805.43)	768590.61 (281174.78 to 1370581.94)	1190.97 (423.26 to 2184.54)	0.16 (0.06 to 0.28)	-2.08 (-2.29 to -1.87)
Henan	1077348.37 (544984.08 to 1665587.10)	1677.89 (818.77 to 2651.09)	1590980.08 (743749.52 to 2620448.46)	1244.16 (558.03 to 2083.12)	0.21 (0.10 to 0.34)	-0.75 (-0.84 to -0.65)
Hong Kong	41914.06 (19736.08 to 66765.80)	733.92 (335.11 to 1191.43)	49992.11 (19796.47 to 92123.35)	360.02 (149.85 to 648.54)	0.19 (0.08 to 0.31)	-2.38 (-2.66 to -2.11)
Hubei	794955.00 (402566.69 to 1247550.61)	1987.60 (958.67 to 3215.89)	779144.18 (300242.90 to 1325080.08)	883.20 (330.02 to 1542.43)	0.18 (0.07 to 0.31)	-2.62 (-2.79 to -2.44)
Hunan	704385.69 (308939.5 to 1190041.24)	1506.62 (624.47 to 2639.40)	1043591.79 (429456.93 to 1782691.77)	1052.20 (431.46 to 1818.12)	0.19 (0.08 to 0.32)	-0.98 (-1.08 to -0.88)
Inner Mongolia	333730.49 (174931.76 to 521199.29)	2369.77 (1165.45 to 3766.85)	560234.41 (283439.71 to 879708.86)	1497.47 (711.67 to 2415.84)	0.23 (0.12 to 0.35)	-1.43 (-1.60 to -1.25)
Jiangsu	622908.29 (300903.68 to 1008237.27)	1094.98 (496.11 to 1792.44)	759173.88 (328370.41 to 1283257.22)	564.24 (244.33 to 956.53)	0.19 (0.08 to 0.31)	-2.63 (-3.46 to -1.79)

Jiangxi	581260.08 (333188.83 to 864808.98)	2260.54 (1250.46 to 3432.01)	579900.73 (281366.69 to 931800.37)	991.56 (464.12 to 1622.19)	0.22 (0.10 to 0.35)	-2.63 (-3.46 to -1.79)
Jilin	573355.82 (325243.36 to 847171.94)	3328.96 (1762.53 to 5021.67)	595749.35 (260034.82 to 986332.69)	1322.43 (557.98 to 2232.51)	0.19 (0.08 to 0.32)	-3.39 (-3.65 to -3.12)
Liaoning	465884.44 (186246.59 to 799497.04)	1495.14 (549.71 to 2628.06)	845289.89 (336984.45 to 1459955.61)	1066.39 (410.11 to 1862.69)	0.17 (0.07 to 0.30)	-0.90 (-1.10 to -0.70)
Macao	2745.20 (1322.35 to 4433.13)	997.20 (473.1 to 1616.85)	4392.71.00 (1947.89 to 7116.76)	442.08 (186.17 to 730.45)	0.20 (0.09 to 0.32)	-2.82 (-2.96 to -2.67)
Ningxia	30506.29 (9699.78 to 58295.59)	1154.14 (336.21 to 2274.61)	70772.33 (25705.48 to 128418.06)	891.44 (295.69 to 1680.20)	0.15 (0.05 to 0.27)	-0.62 (-0.74 to -0.51)
Qinghai	54526.41 (30154.04 to 82891.27)	2053.65 (1049.03 to 3244.71)	111024.00 (57043.06 to 172891.94)	1594.53 (772.28 to 2562.43)	0.23 (0.11 to 0.35)	-0.62 (-0.79 to -0.45)
Shaanxi	472549.21 (237672.78 to 745933.69)	1979.78 (939.40 to 3185.61)	726984.88 (342208.81 to 1179850.91)	1316.37 (600.36 to 2194.50)	0.21 (0.10 to 0.33)	-1.17 (-1.29 to -1.05)
Shandong	1071222.62 (561195.93 to 1720401.21)	1590.68 (792.88 to 2598.74)	1539131.65 (713282.57 to 2532005.13)	997.37 (461.57 to 1670.37)	0.21 (0.10 to 0.33)	-1.51 (-1.57 to -1.45)
Shanghai	144474.17 (67883.86 to 234376.23)	991.42 (437.05 to 1633.62)	165059.36 (70019.30 to 282140.72)	381.45 (159.60 to 662.46)	0.17 (0.07 to 0.29)	-3.59 (-3.89 to -3.29)
Shanxi	298393.43 (107221.54 to 552304.67)	1376.48 (456.49 to 2605.06)	548722.58 (220622.97 to 967829.76)	1076.83 (405.19 to 1971.27)	0.17 (0.07 to 0.30)	-0.78 (-0.91 to -0.66)

Sichuan	1034096.48 (465257.46 to 1743564.68)	1211.92 (508.70 to 2115.74)	1124609.17 (453433.41 to 1983319)	846.99 (336.86 to 1495.25)	0.18 (0.08 to 0.30)	-0.80 (-1.06 to -0.54)
Tianjin	103936.66 (42862.57 to 175464.72)	1335.38 (521.17 to 2313.49)	167433.99 (57598.75 to 303115.84)	745.78 (244.56 to 1384.21)	0.15 (0.05 to 0.27)	-1.77 (-2.15 to -1.39)
Tibet	62413.01 (33451.63 to 95774.01)	4018.05 (2064.38 to 6332.62)	72308.19 (40229.91 to 109043.41)	2440.51 (1274.33 to 3772.27)	0.27 (0.15 to 0.41)	-1.97 (-2.11 to -1.83)
Xinjiang	188840.20 (83695.05 to 314757.70)	1977.04 (798.62 to 3414.63)	511481.92 (268318.86 to 804495.29)	1877.72 (934.52 to 3041.33)	0.24 (0.12 to 0.37)	0.07 (-0.08 to 0.23)
Yunnan	355953.24 (154167.45 to 595292.57)	1351.70 (554.38 to 2325.10)	654473.53 (289548.54 to 1098343.44)	1115.25 (467.87 to 1907.43)	0.19 (0.08 to 0.31)	-0.44 (-0.65 to -0.23)
Zhejiang	479190.35 (274410.67 to 723710.36)	1987.60 (958.67 to 3215.89)	545047.42 (288910.64 to 846279.27)	614.14 (318.24 to 961.53)	0.24 (0.13 to 0.37)	-2.79 (-2.94 to -2.65)

DALYs=disability-adjusted life years; ASDR=age-standardized DALYs rate; PAF=population attributable fraction; EAPC=estimated annual percentage change.
